# Kentucky State Medical Society

**Published:** 1881-06

**Authors:** 


### KENTUCKY STATE MEDICAL SOCIETY.

Twenty-sixth Annual Session, held at Covington, April 5, 6, and 7, 1881.

The Society convened at twelve o'clock, on Tuesday, April 5th, with the president, Dr. L. Beecher Todd, of Lexington in the chair. After the reading of the minutes, and the usual reports of Secretary and Treasurer and the Chairman of the committee of Arrangements, the programme was entered upon.

*Report on Surgery.*—The report on Improvements in Surgery was made by Dr. Fayette Dunlap, of Danville, who, by way of introducing the subject, said that while there had been no want of zeal in the advancement of surgical science during the past year, no startling discovery or strikingly new procedure had been established in practice. The interest recently manifested in the surgery of the kidney was mentioned, and the belief expressed that many conditions now regarded as irremediable will in the early future, be benefited, if not cured by timely surgical interference. The elastic bandage in the treatment of aneurisms of the extremities is advancing in favor, and though recorded experience is limited, it promises much in the treatment of this class of aneurisms. Excision of joints and bones for the correction of deformities, whether in the rachitic or robust, congenital or acquired, has given better results than ever before, and has been largely practiced. It is claimed that the splendid results of this branch of conservative surgery is to be attributed to the antiseptic method of Lister. He called attention to the splendid record of Bigelow's operation, and said it was now firmly established in practice, and its introduction marks an era in the surgery of calculous disease. Experience has fully sustained every claim of its author, and the reliability of his maxims is established. He next alluded to the introduction of Chian turpentine as a remedy for cancer, and said that, after being extensively applied to the treatment of this form of disease, it had been laid aside as useless, either as palliative or curative of cancerous affections. The long-hoped-for discovery of a curative agent in malignant disease finds no encouragement in this drug, and it will soon be forgotten.

Much attention has been directed to Freund's method of extirpation of the uterus, in whole or in part, and though it has met with some favor, and given good results in the hands of a few, its chances for becoming widely adopted are by no means favorable. Its performance is now relegated to a few skilful gynecologists, and there is very little hope of any increase of the favor now extended to the operation.

The antiseptic system in surgery was given extensive notice, and its present status quite thoroughly defined. While no marked progress could be claimed for Listerism during the past year, it certainly had lost nothing in favor, and is as enthusiastically advocated and as zealously practiced by its distinguished founder and his disciples as at any previous time. The system is practiced wherever advanced ideas and methods have reached, and certainly those who look upon it with scepticism are becoming fewer in number. The opposition to the method is small, and while not adopted in all its details everywhere, very few surgeons fail to take advantage of its main features. It has brought before the profession the entire subject of the treatment of wounds, and has certainly taught some valuable lessons. Like everything emanating from Mr. Lister, his recent lecture on the value of the catgut ligature promises to create a decided impression in favor of the application of animal ligatures. His claims are strengthened by experiments and practical experience, and come, consequently, with force. The reintroduction of the animal ligature in a greatly improved form was announced as one of the important events of the last year in surgical practice.

*Report on Practice of Medicine.*—Dr. Charles H. Thomas, of Covington, next read a report on the Improvements in Practical Medicine. He treated, *in extenso*, the subject of the germ theory in reference to acute infectious diseases. He stated that the belief is extending that pneumonia is dependent upon and originates in a specific cause, and that this cause is independent of malaria. He favored the expectant method of treatment in this disease, and said the specific influence of quinine in its management was worthy of thoughtful consideration. In that type of the disease where the temperature is excessively and dangerously high, he favored the exhibition of antipyretic agents, particularly quinine. He then outlined recent investigations tending to establish the infectious character of tuberculosis ,calling especial attention to the fact that it had been shown that the disease may be communicated by the milk and flesh of tuberculous animals.

Dr. Pinckney Thompson, as Chairman of the Committee on Hygiene, made an elaborate and carefully prepared report. This report was for the most part devoted to a detailed account of the present status of sanitary science in Kentucky, the exisiting legislation relating to the public health, and the necessity of additional legislation, so as to bring the State laws up to the standard of advanced ideas in these matters. The report concluded with a delineation of the relations of the medical profession to sanitary legislation.

*The better Protection of the Insane.*—The address of the president, Dr. L. Beecher Todd, of Lexington, was delivered in the evening, thus closing the first day's session. The address was devoted to topics of urgent interest just now to the profession. He alluded to the present system of appointing medical officers to our insane asylums as rewards for party work and personal preference as follows:

The better protection of the insane appeals to you, my fellows, as well as to the politician and political economist, to labor and legislate for their relief. That relief is asked for through you of the Kentucky State Society this evening from those at our very doors in whose behalf I most earnestly appeal to you. That relief is to be obtained only by legislative enactment taking from the governor of the commonwealth the power of appointing medical superintendents for the hospitals for the insane, and returning the power of appointing that officer to the boards of commissioners, where it belongs, and whence it was taken but a few years ago. These commissioners should receive adequate compensation for the faithful performance of the delicate and responsible duties; they should be men of high character for learning, integrity, and philanthropy, eschewing all local and other prejudices, but above all and especially *politics*, that fatal rock against which the hopes and fortunes of many of our charitable institutions have been dashed and wrecked. The medical superintendent should hold office during life or good behavior, the question of the latter to be decided by a court composed of all the commissioners of the State. . . . Frequent change of medical superintendents must needs be detrimental to restoration of patients and general prosperity of the institutions. Therefore this relief to the unfortunate is demanded by humanity and the progress of the age. In the name and for the sake of those who wander forth with the gates of reason closed behind them, I demand it. Let the Kentucky State Medical Society demand it, and with such a respectful emphasis and firm authority that no man standing for office can look his constitutufency in the face and dare refuse it.

SESSION OF SECOND DAY—WEDNESDAY.

After the hour for irregular and executive business had elapsed, Dr. J. W. Holland, of Louisville, read a paper on

*Plumbism from the use of Cosmetics.*

Dr. Holland called attention to the fact that there are certain distinctive, though rather vague symptoms of lead-poisoning which precede the more marked symptoms of wrist-drop, colic, and le[illegible]d-line, and which, when more carefully studied, would suffice to lead an earlier diagnosis. These symptoms he described as headache, vertigo, slight colicky pains, and constipation. He then gave in detail an illustrative case of a woman who, two years before coming under observation, had begun the use of flake-white powder as a cosmetic. After exhibiting the symptoms already mentioned, she had an attack of melancholia of a month's duration, afterward the signs of plubism—double wrist drop and the blue line on the gums—were abruptly presented. He related in detail several similar cases illustrating the essential points deduced from the paper; that lead may be introduced into the system to the extent of its toxic effects when applied to the skin in the form of powder and lotions; that the most popular beautifying cosmetics contain lead. The results of the chemical analysis of various popular cosmetics were given in detail.

Dr. A. M. Vance, of Louisvi le, read a paper upon

*An Improved Dressing for Spinal Caries and Curvature.*

He stated that three years since he addressed the society upon this subject, and he now purposed to give the result of a very considerable study of this class of dressings in the treatment of spinal curvature. The dressing consists of paper, glue, and steel stays, so arranged and fitted as to meet all the indications of the plaster-of-Paris jacket, and at the same time permit its removal and reapplication. The additional advantages claimed for the dressing are the qualities of lightness, firmness, perviousness, and cheapness. He exhibited the dressing in several sizes, one specimen having the jury-mast attached, and detailed the particulars of its construction and method of application. He reported a number of cases in which he had successfully used the dressing.

Dr. W. W. Dawson, of Cincinnati, in response to the invitation of the president, spoke at some length upon the subject of splints and the treatment of Potts' disease of the spine. He said the entire subject of fixed dressings was one of great importance, and though much has been accomplished by the plaster-of-Paris jacket in spinal disease, much yet remaius to be accomplished before the ideal dressing for relieving this condition can be attained. The qualities desired are elasticity sufficient for motion, together with unyielding support to the diseased vertebra—qualities which, from the nature of things, are most difficult of combination. He said he was not sure that he had ever seen the spine when in this condition of the disease unbent or straightened by suspension or the application of the jacket. He does not believe that the angle of curvature could be changed, from the fact that the altered condition of bone and ligaments would not permit it. Hence, he always considered the measurements with the lead tape, as made by Dr. Sayre, to be deceptive. He said he had satisfied himself, in treating Pott's disease of the spine, with arresting the disease at the stage presented when the treatment was instituted, without attempting to reduce existing deformity. As a means of extension he preferred the hammock introduced by Mr. Richard Davy, of London, rather than the suspension method. In conclusion, he expressed the belief that the dressings now in use were too rigid, and said the great desideratum was an appliance that would give the same amount of support and be easy and yielding.

Dr. D. W. Yandell said this is a department of surgery in which we have every reason to expect much advance and great improvement. He, too, is quite satisfied with the present means at command, to lock the deformity just where he finds it and leave it alone. He related an instance in Louisville where an instrument-maker attempted by screws and steel to correct a deformity when anchylosis was progressing well, and the death of the child followed. As a means of extension he prefers simple extension by the hands to either suspension by pulleys or in the hammock. He has, however, almost abandoned extension, on account of the danger of disturbing the anchylosis. The plaster-of Paris he considers the best dressing to secure rest and immobility while anchylosis is in progress, and after that time he prefers the dressing exhibited by Dr. Vance. In lateral curvature the paper dressing he regarded preferable from the beginning of the treatment, as it could be removed and the patient go through with the gymnastic exercise.

Dr. Dawson called attention to the essential difference in the nature of the lateral and antero posterior curvatures of the spine. The former being due to a want of equilibrium on the part of the muscles, while the latter is due to changes in the bones.

The discussion was continued at some length by Dr. Charles Mann, Dr. W. A. Roberts, Dr. A. M. Vance, and Dr. S. N. McCormack.

